# Characteristics of sarcopenia by European consensuses and a phenotype score

**DOI:** 10.1002/jcsm.12507

**Published:** 2019-12-21

**Authors:** Il‐Young Jang, Eunju Lee, Heayon Lee, Hyungchul Park, Sunyoung Kim, Kwang‐il Kim, Hee‐Won Jung, Dae Hyun Kim

**Affiliations:** ^1^ Department of Internal Medicine, Asan Medical Center University of Ulsan College of Medicine Seoul Republic of Korea; ^2^ PyeongChang Health Center and County Hospital PyeongChang Gangwon‐Do Republic of Korea; ^3^ Department of Family Medicine Kyung Hee University Medical Center Seoul Republic of Korea; ^4^ Division of Geriatrics, Department of Internal Medicine Seoul National University Bundang Hospital Republic of Korea; ^5^ Department of Internal Medicine Seoul National University Hospital Seoul Republic of Korea; ^6^ Marcus Institute for Aging Research Hebrew Senior Life Boston MA USA; ^7^ Division of Gerontology, Department of Medicine Beth Israel Deaconess Medical Center Boston MA USA

**Keywords:** Sarcopenia, Frailty, Validation, Outcome, Prospective study

## Abstract

**Background:**

We aimed to assess the clinical characteristics of sarcopenia by the original and revised European Working Group on Sarcopenia in Older People (EWGSOP 1 and 2), and to propose a new sarcopenia phenotype score (SPS) to improve relevance of clinical outcomes.

**Methods:**

Analyses were performed in 1408 older adults of the Aging Study of PyeongChang Rural Area, a community‐based cohort in Korea. For sarcopenia definitions, we used EWGSOP 1, EWGSOP 2, and SPS, a new index counting number of abnormal domains among components of grip strength, gait speed, or muscle mass. Frailty status by the frailty index and the Cardiovascular Health Study frailty score was compared with sarcopenia measures. Prediction ability for composite outcome combining death and institutionalization due to functional decline was assessed among sarcopenia measures.

**Results:**

Generally, sarcopenia spectrum by both EWGSOP 1 and 2 was associated with worse functional status in parameters of geriatric assessments. However, population who were considered as sarcopenic by EWGSOP 1, but not by EWGSOP 2, showed increased risk of composite outcome and worse frailty status, compared with people who were classified as not sarcopenic by both EWGSOP 1 and 2. With SPS, dose–response relationship was observed with both frailty status and outcome prediction. Prediction for composite outcome was better in SPS than in EWGSOP 2 classification.

**Conclusions:**

A new SPS might be used to classify sarcopenic burden in older adults to resolve possible inconsistencies in phenotype correlation and outcome prediction of EWGSOP 2 criteria.

## 1 Introduction

In older adults, appropriate muscle strength and power are essential to independently perform activities in daily life.^1^, ^2^, ^3^ Decreased muscle mass, strength, or power, known as sarcopenia, is a common condition as anabolic resistance and catabolic pressure in maintaining muscle homeostasis increases with aging in older adults.^4^, ^5^ Sarcopenia is associated with falls, functional impairment, and mortality in community‐dwelling older adults.^3^, ^6^, ^7^, ^8^ Because muscle mass and function reflect physiological reserve, these parameters also associated with poor outcomes in patients receiving stressful treatments, such as cancer chemotherapy, organ transplantation, and cardiovascular procedures.^9^, ^10^, ^11^, ^12^, ^13^, ^14^ Based on these evidences, the Centers for Medicare and Medicaid Services and the National Center for Health Statistics included sarcopenia as a disease in the *International Classification of Diseases, 10th Revision, Clinical Modification* in 2016.^15^

To prevent adverse outcomes associated with sarcopenia, it is critical to standardize definition of sarcopenia for screening and diagnosis. Because muscle mass, strength, and physical performance are affected by demographic and anthropometric characteristics, several population‐specific consensus guidelines have been developed, such as the European Working Group on Sarcopenia in Older People (EWGSOP 1),^7^ the Foundation for the National Institutes of Health,^16^ and the Asian Working Group of Sarcopenia guidelines.^17^ These guidelines have evolved to reflect up‐to‐date understanding about sarcopenia. In earlier studies by Janssen *et al*.^18^ and Baumgartner *et al*.^19^, sarcopenia was defined using decreased muscle mass alone. The EWGSOP 1 used muscle mass, strength, and physical performance, recognizing that muscle function is not solely determined by muscle mass.^7^ The latest European consensus (EWGSOP 2) emphasized clinical suspicion and muscle strength,^20^ based on the evidence that muscle function is more important than muscle mass in predicting clinical outcomes.^21^, ^22^ However, clinical implications of these updates in sarcopenia guidelines have not been investigated.

In this study, we examined the clinical characteristics and outcomes associated with sarcopenia defined using EWGSOP 1 vs. EWGSOP 2. In addition, we aimed to propose an alternative sarcopenia phenotype score (SPS) based on components of EWGSOP 1 and 2 to improve prediction of clinical outcomes.

## 2 Materials and methods

### 2.1 Study population

The Aging Study of PyeongChang Rural Area (ASPRA) is a large, community population‐based, prospective cohort study of aging in rural areas. The detailed design and methods of ASPRA have been described elsewhere.^23^ Briefly, community‐dwelling adults aged 65 years or older in PyeongChang Country, Gangwon Province, Korea, located 180 km east of Seoul, were enrolled and underwent annual geriatric assessments of medical, physical functional, and psychosocial status.

In this study, we assessed data from 1446 individuals aged 65–101 years who had baseline examination from December 2014 to July 2018. Of these, we excluded 38 who had missing muscle mass data, leaving 1408 participants included in this analysis. All participants provided written informed consent. The institutional review board at Asan Medical Center, Seoul, Korea, approved the study. All authors of this manuscript comply with the guidelines of ethical authorship and publishing in the *Journal of Cachexia, Sarcopenia and Muscle*.^24^

### 2.2 Assessment of sarcopenia

We measured muscle mass, grip strength, and usual gait speed to define sarcopenia with EWGSOP 1 and 2. Muscle mass was measured by the bioelectrical impedance analysis (InBody 620; InBody, Seoul, Republic of Korea) with measuring frequencies of 5, 50, and 500 kHz. After overnight fasting to reduce the error, the four limbs impedance was measured at the standing position using the device for quantification of body composition, including total mass and lean mass. Appendicular skeletal muscle (ASM) was calculated by summing the lean mass of both arms and legs. ASM was adjusted by height squared (ASM/ht^2^) to compare muscle mass between participants. In this study, low muscle mass was defined as ASM/ht^2^ < 7.0 kg/m^2^ for men and < 6.0 kg/m^2^ for women. Grip strength was assessed using a handgrip dynamometer (T.K.K 5401 Grip‐D; Takei, Tokyo, Japan) in the sitting position with the elbow flexed. The maximal value of two measurements in the dominant arm taken at least 1 min interval was recorded. In this study, low grip strength was defined as the maximal grip strength < 27 kg for men and < 16 kg for women. For usual gait speed, participants were instructed to walk 7 m in their usual walking speed on a flat indoor surface. Trained nurses measured the transit time of 4 m by a digital stopwatch, excluding an acceleration and deceleration interval of 1.5 m. The usual gait speed was calculated from the time taken to walk 4 m (meters per second). Slow gait was defined as < 0.8 m/s.

In EWGSOP 1, sarcopenia was diagnosed if the participant had low muscle mass with either low gait speed or low grip strength. In EWGSOP 2, sarcopenia was diagnosed if the participant had low muscle mass and low grip strength. Severe sarcopenia was defined as the presence of slow gait speed, with low muscle mass and low grip strength. Also, as an approach to better stratify the incremental sarcopenic burden of older adults with outcome relevance, we developed a novel SPS that assigns 0 (absence) or 1 (presence) of low muscle mass, decreased grip strength, and slow gait speed, with a total score ranging from 0 to 3.

### 2.3 Assessment of other geriatric conditions

Comprehensive geriatric assessment was performed once a year by trained nurses. We recorded the years of education completed and defined low socio‐economic status as receiving medical aid due to monthly income of less than 500 USD. The short physical performance battery score, ranged from 0 to 12, was calculated from three components: the ability to stand for up for 10 s with feet positioned (together side‐by‐side, semi‐tandem, and tandem), transit time for a 4 m walk, and the chair standing test measuring time required from sit to stand for five times.^25^ We measured the Cardiovascular Health Study (CHS) frailty phenotype and a 34‐item frailty index constructed using Rockwood's deficit accumulation approach.^26^, ^27^, ^28^ We defined disability as the presence of 1 or more impairments in 7 activities of daily living (ADLs) (bathing, continence, dressing, eating, toileting, transferring, and washing face and hands) and 10 instrumental activities of daily living (IADLs) (food preparation, household chores, going out a short distance, grooming, handling finances, laundry, managing own medications, shopping, transportation, and using a telephone).^29^ Multimorbidity was defined as having 2 or more of the 11 physician‐diagnosed illnesses including angina, arthritis, asthma, cancer, chronic lung disease, congestive heart failure, diabetes, heart attack, hypertension, kidney disease, and stroke. We assessed depressive symptoms using the Korean version of the Center for Epidemiological Studies ‐Depression Scale (CES‐D),(range 0–60) with depression defined if CES‐D score ≥ 21.^30^ We used the Mini‐Mental State Examination for Dementia Screening (MMSE‐DS) for cognitive status (range 0–30), with cognitive dysfunction defined if MMSE‐DS score < 24.^31^ We defined the risk for malnutrition if the Mini‐Nutritional Assessment Short Form score was ≤ 11.^32^ Polypharmacy was defined as regularly taking five or more prescription medications.^23^

### 2.4 Outcome measurements

A composite endpoint of death or institutionalization due to functional impairment was assessed. All participants or their family members were interviewed by telephone every 3 months and in‐person annually to obtain vital status or institutionalization for longer than 3 months. Incident disability was defined as the presence of ADL or IADL disability during follow‐up among those without disabilities at baseline.

### 2.5 Statistical analysis

Continuous and categorical variables were compared using one‐way analysis on variance or chi‐square test among those who met both EWGSOP 1 and 2 definitions, the EWGSOP 1 definition alone, and none. We assessed the association between sarcopenic status and frailty measures using *R* ^2^ from linear regression and post hoc analysis using *t*‐tests. Kaplan–Meier cumulative incidence plotting with log‐rank test was used to compare the outcome relevance of sarcopenia criteria. Thereafter, we used Cox proportional hazards models to examine the association of sarcopenia with the composite endpoint. In comparing outcome predictability of SPS and EWGSOP 2 definition, Harrell's C index was used.^33^ A two‐sided *P*‐value <0.05 was considered to be statistically significant, and all analyses were performed using STATA 15.0 (StataCorp, College Station, TX, USA).

## 3 Results

### 3.1 Baseline characteristics

**Figure 1:**
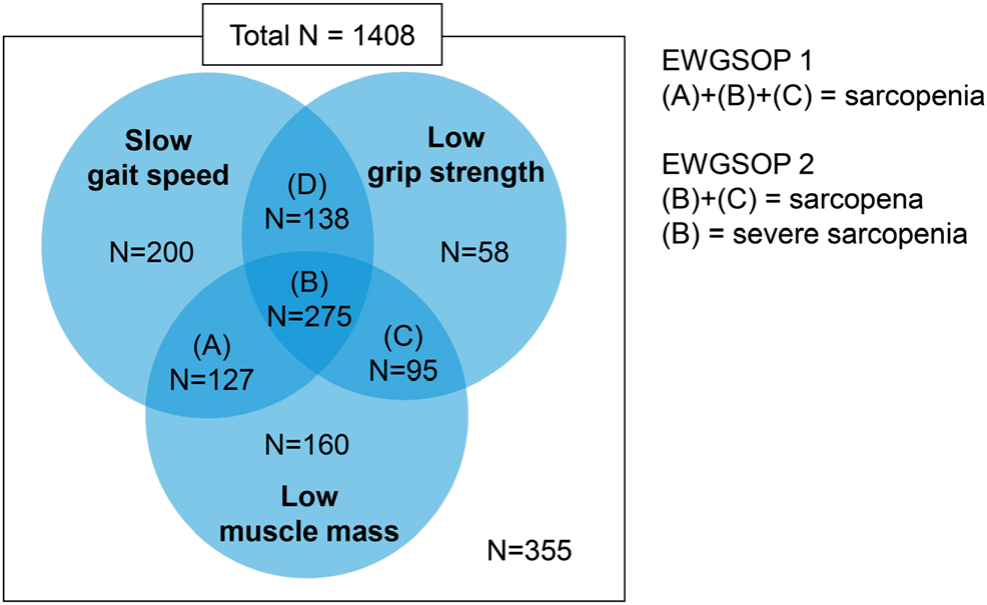
Comparisons on classification of sarcopenia by original (EWGSOP 1) and revised (EWGSOP 2) European consensus for sarcopenia. EWGSOP, European Working Group on Sarcopenia in Older People.

Of 1408 participants, 370 met both EWGSOP 1 and 2 definitions of sarcopenia, 127 met the EWGSOP 1 definition alone, and 911 did not meet any definitions (*Figure* 1). Sarcopenic older adults by either EWGSOP 1 or EWGSOP 2 were older, had shorter duration of education, and with lower body mass index and appendicular skeletal muscle mass (ASM/ht^2^) (*Table* 1). Participants with sarcopenia by either EWGSOP 1 or EWGSOP 2 showed worse physical performance in terms of gait speed, grip strength, or short physical performance battery score. Furthermore, sarcopenic state was associated with disabilities, cognitive impairment, and depressive mood.

**Table 1:** Participants' characteristics according to sarcopenic status, by original (EWGSOP 1) and revised (EWGSOP 2) European consensus for sarcopenia

	Sarcopenia status by (EWGSOP 1/EWGSOP 2)			*P*‐value
	(−/−)	(+/−)	(+/+)	
	*n* = 911	*n* = 127	*n* = 370	
Age	74.1 (5.7)	77.4 (4.8)	81.2 (6.6)‡	< 0.001
Duration of education (years)	5.8 (3.8)	4.4 (2.6)	4.2 (2.5)	< 0.001
Low SES, < 500 USD/m	46 (5.1%)	6 (4.7%)	33 (8.9%)	0.073
BMI (kg/m^2^)	25.6 (3.3)	23.3 (3.2)	22.8 (2.9)	< 0.001
ASM/ht^2^ (kg/m^2^)	6.95 (1.07)	5.60 (0.96)	5.45 (0.89)	< 0.001
Gait speed (m/s)	0.89 (0.30)	0.59 (0.18)	0.63 (0.25)	< 0.001
Grip strength (kg)	26.1 (9.4)	23.0 (6.8)	14.5 (5.3)‡	< 0.001
SPPB score	9.52 (2.46)	7.29 (2.56)	6.77 (3.12)	< 0.001
ADL disability	84 (9.2%)	16 (12.6%)	83 (22.4%)	< 0.001
IADL disability	201 (22.1%)	46 (36.2%)	181 (48.9%)*	< 0.001
Risk of malnutrition by MNA	229 (25.1%)	68 (53.5%)	224 (60.5%)	< 0.001
Cognitive impairment by MMSE‐DS	163 (17.9%)	49 (38.6%)	172 (46.7%)*	< 0.001
Depressive mood by CES‐D	51 (5.6%)	18 (14.2%)	61 (16.6%)	< 0.001
Polypharmacy, ≥ 5 medications	177 (19.4%)	28 (22.1%)	110 (29.7%)	0.006
Fall history, previous year	106 (11.6%)	18 (14.2%)	71 (19.2%)	0.520

Data presented as mean (standard deviation) or number (%). ADL, activities of daily living; ASM/ht^2^, appendicular skeletal m muscle mass adjusted by height square; BMI, body mass index; CES‐D, the Center for Epidemiological Studies depression scale; EWGSOP, European Working Group on Sarcopenia in Older People; IADL, instrumental activities of daily living; MMSE‐DS, the Mini‐Mental State Examination for Dementia Screening; MNA, the Mini Nutritional Assessment Short Form; SES, socio‐economic status; SPPB, short physical performance battery.

* *P*‐value < 0.05 between the middle and the right most column.

† *P*‐value < 0.01 between the middle and the right most column.

‡ *P*‐value < 0.001 between the middle and the right most column.

People who were reclassified as not sarcopenic by EWGSOP 2 but sarcopenic by EWGSOP 1 were younger (*P* < 0.001), with higher grip strength (*P* < 0.001), with less IADL disability (*P* = 0.030), and had less cognitive impairment (*P* < 0.001) than people who met both EWGSOP 1 and 2 definitions.

### 3.2 Sarcopenia status and frailty measures

We sought for the correlation between sarcopenia severities by EWGSOP 1, EWGSOP 2, or SPS and two frailty models—(i) phenotype frailty of CHS and (ii) frailty index of deficit accumulation model (*Table* 2). By EWGSOP 1, frailty index and CHS frailty score were higher in people with sarcopenia, when compared with people with no sarcopenia. According to EWGSOP 2, frailty index and CHS frailty score were trended to be higher with worsening state of sarcopenia (*P* for trend <0.001).

**Table 2:** Comparison of the original (EWGSOP 1) and revised (EWGSOP 2) European consensus for sarcopenia and the new sarcopenia phenotype score (SPS) for cross‐sectional correlations with the Cardiovascular Health Study (CHS) frailty score and frailty index

	CHS frailty score	*P*‐value	Frailty index	*P*‐value
EWGSOP 1				
No sarcopenia	1.07 (1.11)	(ref)	0.15 (0.10)	(ref)
Sarcopenia	2.35 (1.00)	< 0.001	0.26 (0.12)	< 0.001
EWGSOP 2				
No sarcopenia	1.18 (1.13)	(ref)	0.16 (0.10)	(ref)
Non‐severe sarcopenia	1.54 (0.74)	0.002	0.19 (0.07)	0.002
Severe sarcopenia	2.83 (0.82)	< 0.001	0.31 (0.12)	< 0.001
EWGSOP 1/2				
No sarcopenia by both EWGSOP 1 and 2	1.07 (1.11)	(ref)	0.15 (0.10)	(ref)
Sarcopenia by only EWGSOP 1	1.93 (0.96)	< 0.001	0.21 (0.11)	< 0.001
Sarcopenia by both EWGSOP 1 and 2	2.50 (0.98)	<0.001	0.27 (0.12)	< 0.001
SPS (sarcopenia phenotype score)				
0	0.31 (0.55)	(ref)	0.10 (0.07)	(ref)
1	1.19 (0.96)	< 0.001	0.15 (0.10)	< 0.001
2	2.11 (0.95)	< 0.001	0.22 (0.10)	< 0.001
3	2.83 (0.82)	< 0.001	0.30 (0.12)	< 0.001

Data presented as mean (standard deviation). *P*‐values were calculated by independent *t*‐test with reference status for each sarcopenia spectrum, with no consideration for multiple comparisons. EWGSOP, European Working Group on Sarcopenia in Older People; ref, reference.

Thereafter, we performed a subgroup analysis in 1038 participants with no sarcopenia by EWGSOP 2. In this subpopulation, mean frailty index was higher (*P* < 0.001) in people who were sarcopenic when classified by EWGSOP 1 [mean 0.21, standard deviation (SD) 0.11, *n* = 127] than those who were not sarcopenic by EWGSOP 1 (mean 0.15, SD 0.10, *n* = 911). Similarly, positive count of the CHS frailty phenotype was higher in people who were classified as sarcopenic by EWGSOP 1 but not by EWGSOP 2 (mean 1.93, SD 0.96, *n* = 127) than people who were classified as not sarcopenic by both EWGSOP 1 and 2 (mean 1.07, SD 1.11, *n* = 911).

We assessed for distributions of phenotype frailty and frailty index according to SPS. Of 1408 participants, 355 (25.2%), 418 (29.7%), 360 (25.6%), and 275 (19.5%) had SPS of 0, 1, 2, and 3 points, respectively. The mean counts of CHS frailty phenotype were 0.31 (SD 0.55), 1.19 (SD 0.96), 2.11 (SD 0.95), and 2.83 (SD 0.82) for SPS of 0, 1, 2, and 3 points, respectively (*P* for trend < 0.001, *R* ^2^ = 0.54). The mean frailty indices were 0.10 (SD 0.07), 0.15 (SD 0.10), 0.22 (SD 0.10), and 0.30 (SD 0.12) for SPS of 0, 1, 2, and 3 points, respectively (*P* for trend < 0.001, *R* ^2^ = 0.35).

### 3.3 Sarcopenic status and the incidence of death or institutionalization

During a mean follow‐up of 29.7 months (SD 12.6), 47 people died, and 99 people were institutionalized to long‐term care hospitals or nursing facilities.

**Figure 2:**
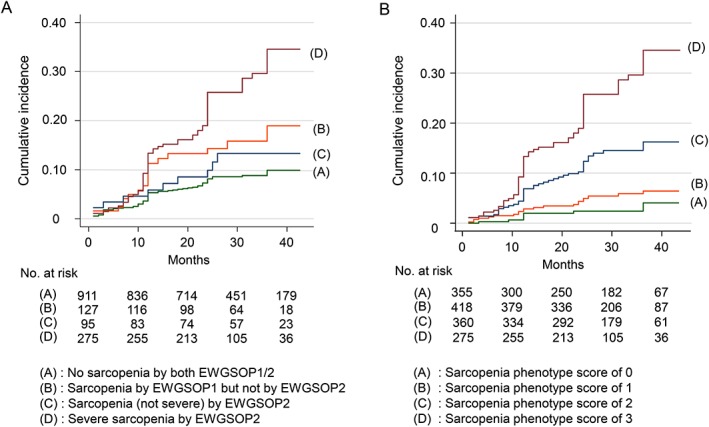
Kaplan–Meier cumulative incidence plot for geriatric adverse outcome including mortality and institutionalization during observation period, by baseline sarcopenic status. (*A*) Original (EWGSOP 1) and revised (EWGSOP 2) European consensus for sarcopenia—(A) no sarcopenia by either EWGSOP 1 or EWGSOP 2; (B) sarcopenia by EWGSOP 1 but not considered as sarcopenic by EWGSOP 2; (C) sarcopenia, not severe, by EWGSOP 2; and (D) severe sarcopenia by EWGSOP 2 (log‐rank *P* < 0.001). (*B*) newly proposed sarcopenia phenotype score—(A) sarcopenia phenotype score of 0; (B) sarcopenia phenotype score of 1; (C) sarcopenia phenotype score of 2; and (D) sarcopenia phenotype score of 3/3 (log‐rank *P* < 0.001). EWGSOP, European Working Group on Sarcopenia in Older People.

Sarcopenic status by EWGSOP 1 and severe sarcopenia by EWGSOP 2 were associated with an increased rate of composite endpoint, even after adjusting for age, gender, baseline ADL disabilities, and multimorbidity (*Table* 3). However, non‐severe sarcopenia by EWGSOP 2 (preserved gait speed) was not significantly associated with the composite outcome. When classifications of EWGSOP 1 and EWGSOP 2 were compared for outcomes, sarcopenia by EWGSOP 1 classification but not by EWGSOP 2 (specifically, people with spared grip strength intact, low gait speed, and low muscle mass) was associated with increased probability of mortality or institutionalization, even after adjusting for age, gender, baseline ADL disabilities, and multimorbidity (*Table* 3). *Figure* 2A shows cumulative incidence of the composite endpoint according to baseline sarcopenic status by EWGSOP 1 and 2 (log‐rank test *P* < 0.001).

**Table 3:** Comparison of the original (EWGSOP 1) and revised (EWGSOP 2) European consensus for sarcopenia and the new sarcopenia phenotype score (SPS) in predicting a composite endpoint of mortality and institutionalization due to functional impairment

	Model 1		Model 2		Model 3	
	HR	95% CI	HR	95% CI	HR	95% CI
EWGSOP 1						
No sarcopenia	(ref)					
Sarcopenia	3.76	2.66–5.31	2.29	1.56–3.36	2.19	1.50–3.22
EWGSOP 2						
No sarcopenia	(ref)					
Non‐severe sarcopenia	1.60	0.82–3.10	1.09	0.56–2.14	1.14	0.58–2.23
Severe sarcopenia	3.99	2.85–5.59	2.04	1.38–3.04	1.92	1.29–2.86
EWGSOP 1/2						
No sarcopenia by both EWGSOP 1 and 2	(ref)					
Sarcopenia by only EWGSOP 1	2.80	1.65–4.77	2.54	1.48–4.35	2.46	1.43–4.21
Sarcopenia by both EWGSOP 1 and 2	4.10	2.86–5.87	2.20	1.47–3.32	2.11	1.40–3.17
SPS (sarcopenia phenotype score)							
0	(ref)						
1	1.67	0.78–3.54	1.45	0.68–3.09	1.38	0.64–2.94	
2	4.18	2.11–8.27	3.00	1.48–6.10	2.79	1.37–5.68	
3	8.70	4.48–16.90	4.37	2.11–9.05	3.86	1.85–8.03	

Model 1: unadjusted; Model 2: age, gender adjusted; and Model 3: age, gender, baseline disability, multimorbidity adjusted. CI, confidence interval; EWGSOP, European Working Group on Sarcopenia in Older People; HR, hazard ratio; ref, reference.

Observing inconsistent associations of sarcopenic spectrum classified by EWGSOP 1 and 2 criteria with the composite endpoint, we analysed the outcome relevance of sarcopenic burden using the novel SPS. We found a dose–response relationship between SPS and the composite endpoint (*Figure* 2B and *Table* 3). Also, prediction ability for the composite outcome was higher (*P* < 0.001) for SPS (C statistic 0.71, 95% confidence interval 0.67– 0.75) than for EWGSOP 2 spectrum of no sarcopenia, non‐severe sarcopenia, and severe sarcopenia (C statistic 0.65, 95% confidence interval 0.61– 0.69).

## 4 Discussion

In this prospective cohort‐based study on geriatric outcomes according to baseline sarcopenia status by EWGSOP 1, EWGSOP 2, and SPS, we found that the sarcopenia spectrum is associated with adverse outcomes in a dose‐responsive manner in Korean rural community‐dwelling older adults. Even though sarcopenia status in general by either EWGSOP 1 or EWGSOP 2 was significantly associated with the incidence of adverse outcomes in the future, non‐severe sarcopenia by EWGSOP 2 was insignificant in terms of clinical outcomes. Also, people who were classified as sarcopenic by EWGSOP 1 and then reclassified as not sarcopenic by EWGSOP 2 showed increased risk of combined mortality and institutionalization due to functional decline in the future. To resolve the mismatch between clinical outcomes and current guidance on sarcopenia classification, we implemented a new phenotype model of sarcopenia (SPS) using similar components of EWGSOP 1 and 2 and found that this scoring approach could produce a stepwise, dose–response predictability for adverse outcomes.

In the history of researches on sarcopenia, there have been debates on how to define sarcopenia, with varying clinical parameters for muscle mass, strength, and physical performance. Since Rosenberg coined the term ‘sarcopenia’ technically meaning deficiency of muscle mass, numerous studies have tried to address clinical and social relevance of sarcopenia in older adults 34, 35, 36 and also tried to provide guidance to tackle this progressive, self‐aggravating geriatric syndrome with interventional efforts.^37^ The guidelines for screening sarcopenia have evolved around the effort to detect older adults who may benefit from population‐wise prevention efforts. In EWGSOP 2, sarcopenia was considered as a clinical condition of spectrum leading to disability, calling for initiating in‐depth clinical assessments and interventions for people deemed as probable sarcopenia in the decision tree of the guideline.^3^

One of the notable points of the EWGSOP 2 is excluding people who have spared muscle strength (by grip strength test or chair stand test) in the decision process of assessments on sarcopenia. However, in our prospective research on a community population, increased adverse outcome was still observed in people with spared grip strength but decreased muscle mass and gait speed. In our study population, there were substantial number (127, 9.0% of the study population) of individuals who had spared grip strength, but with low muscle mass and gait speed, who were initially considered sarcopenic in EWGSOP 1, but not considered sarcopenic in EWGSOP 2. Conversely, people with non‐severe sarcopenia by EWGSOP 2 did not have significantly increased risk for adverse outcomes, compared with people who were classified as not sarcopenic in EWGSOP 2. On the other hand, when the number of abnormal domains of sarcopenia components was counted, baseline SPS showed incremental risk probabilities for incidental geriatric outcomes. With extensive research evidences showing gait speed or physical performance as an important risk for future functional decline and mortality in older adults,^38^, ^39^, ^40^ we suggest that people with spared grip strength, low gait speed, and decreased muscle mass might be regarded as sarcopenic.

In terms of frailty and sarcopenia, authors of EWGSOP 2 suggested that physical frailty and sarcopenia largely overlap but are still distinct and they considered sarcopenia as a disease, while physical frailty as a broad geriatric syndrome. In our study, we observed notable correlations between sarcopenia status and frailty status, by either the CHS phenotype model or frailty index of deficit accumulation model. Average frailty status of people with severe sarcopenia by EWGSOP 2 corresponded to frailty by either CHS phenotype or frailty index.^28^, ^41^, ^42^ This correlation between sarcopenic burden and frailty spectrum was clearer when SPS was compared with frailty phenotype or frailty index, suggesting sarcopenia as a subsystem in the spectrum of frailty occurring through the aging process.

Regarding correlations on frailty and sarcopenic burden in our study population, we found that graded interventional approaches on sarcopenia–frailty spectrum of community‐dwelling older adults might be conceivable. In the population with SPS of 0 to 1 whose geriatric risk probability is low, general primary prevention with health maintenance and screening measures might be sufficient. For people with SPS of 2, corresponding to prefrailty, structured multicomponent community intervention programmes to improve physical performance to ensure functional independence and to prevent possible geriatric clinical outcomes might be beneficial.^37^ In people with SPS of 3, whose 3 years of outcome risk for combined mortality and chronic institutionalization exceeds 30% in our study, comprehensive geriatric assessments to find domains with deficits, efforts to reconcile treatments on multimorbidity and geriatric syndromes, and planning for advanced care may be appropriate.

To the authors' knowledge, this is the first study to assess geriatric clinical outcomes according to EWGSOP 1, EWGSOP 2, and a newly proposed SPS in a large community‐based prospective cohort. Because the ASPRA has its strength in successfully recruiting more than 90% of eligible community‐dwelling population with nearly all population in the region served by the government‐based health care system, we could capture a wide spectrum of sarcopenia status, with less selection bias of participants.^23^

However, there are limitations in our study. In outcome prediction modelling, we used combined institutionalization due to functional decline and mortality as a major study outcome, because mortality alone was insufficient to produce statistically meaningful differences in analysis. However, as a study showing that chronic institutionalization occurs in older adults who are functionally dependent, and rather irreversible, resulting in death in initially admitted facilities,^43^ institutionalization might be considered as a major outcome of mortality. Also, the cohort study was based in a single rural region of Korea; therefore, generalization in the global population may be restricted.

In conclusion, we assessed the geriatric clinical characteristics and outcomes of sarcopenia status defined by EWGSOP 1, EWGSOP 2, and a newly proposed SPS. We found that the sarcopenia burden is highly associated with frailty status and increment probabilities for future adverse outcomes. Future interventional studies on sarcopenia and frailty can use SPS as a guidance to select population who might benefit from tailored intervention programmes for corresponding sarcopenia status.

## Conflict of interest

D.H.K. is a consultant to Alosa Health, a nonprofit educational organization with no relationship to any drug or device manufacturers. The other authors have no potential conflicts of interest to disclose.

## Funding

The Aging Study of PyeongChang Rural Area was supported by PyeongChang County Hospital, Gangwon Province. This study was also supported by a philanthropic fund donated by the Herald through Asan Institute for Life Sciences and Corporate Relations of Asan Medical Center, Seoul, Korea. D.H.K. is supported by grant numbers R01AG056368, P30AG031679, and P30AG048785 from the National Institute on Aging. Public health professionals and nurses of PyeongChang County Hospital participated in data collection. Otherwise, sponsors did not have any role in study design; analysis and interpretation of data; the writing of the article; and the decision to submit the article for publication.

## References

[1] Jang HC . How to diagnose sarcopenia in Korean older adults? Ann Geriatr Med Res 2018;22:73–79. 10.4235/agmr.2018.22.2.73 PMC7387610 32743250

[2] Jang I‐Y , Jung H‐W , Lee CK , Yu SS , Lee YS , Lee E . Comparisons of predictive values of sarcopenia with different muscle mass indices in Korean rural older adults: a longitudinal analysis of the aging study of PyeongChang rural area. Clin Interv Aging 2018;13:91. 29391783 10.2147/CIA.S155619 PMC5769584

[3] Cruz‐Jentoft AJ , Bahat G , Bauer J , Boirie Y , Bruyere O , Cederholm T , et al. Sarcopenia: revised European consensus on definition and diagnosis. Age Ageing 2018;48. 10.1093/ageing/afz046 PMC6593317 31081853

[4] Morley JE . Pharmacologic options for the treatment of sarcopenia. Calcif Tissue Int 2016;98:319–333. 26100650 10.1007/s00223-015-0022-5

[5] Breen L , Phillips SM . Skeletal muscle protein metabolism in the elderly: interventions to counteract the ‘anabolic resistance’ of ageing. Nutr Metab 2011;8:68. 10.1186/1743-7075-8-68 PMC3201893 21975196

[6] Bunout D , de la Maza MP , Barrera G , Leiva L , Hirsch S . Association between sarcopenia and mortality in healthy older people. Australas J Ageing 2011;30:89–92. 21672118 10.1111/j.1741-6612.2010.00448.x

[7] Cruz‐Jentoft AJ , Baeyens JP , Bauer JM , Boirie Y , Cederholm T , Landi F , et al. Sarcopenia: European consensus on definition and diagnosis: report of the European Working Group on Sarcopenia in Older People. Age Ageing 2010;39:412–423. 20392703 10.1093/ageing/afq034 PMC2886201

[8] Cruz‐Jentoft AJ , Landi F , Topinkova E , Michel JP . Understanding sarcopenia as a geriatric syndrome. Curr Opin Clin Nutr Metab Care 2010;13:1–7. 19915458 10.1097/MCO.0b013e328333c1c1

[9] Kahn J , Wagner D , Homfeld N , Muller H , Kniepeiss D , Schemmer P . Both sarcopenia and frailty determine suitability of patients for liver transplantation—a systematic review and meta‐analysis of the literature. Clin Transplant 2018;32:e13226. 29478305 10.1111/ctr.13226

[10] Lee JS , He K , Harbaugh CM , Schaubel DE , Sonnenday CJ , Wang SC , et al. Frailty, core muscle size, and mortality in patients undergoing open abdominal aortic aneurysm repair. J Vasc Surg 2011;53:912–917. 21215580 10.1016/j.jvs.2010.10.111

[11] Englesbe MJ , Patel SP , He K , Lynch RJ , Schaubel DE , Harbaugh C , et al. Sarcopenia and mortality after liver transplantation. J Am Coll Surg 2010;211:271–278. 20670867 10.1016/j.jamcollsurg.2010.03.039 PMC2914324

[12] Jung H‐W , Kim JW , Kim J‐Y , Kim S‐W , Yang HK , Lee JW , et al. Effect of muscle mass on toxicity and survival in patients with colon cancer undergoing adjuvant chemotherapy. Support Care Cancer 2015;23:687–694. 25163434 10.1007/s00520-014-2418-6

[13] Antoun S , Borget I , Lanoy E . Impact of sarcopenia on the prognosis and treatment toxicities in patients diagnosed with cancer. Curr Opin Support Palliat Care 2013;7:383–389. 24189893 10.1097/SPC.0000000000000011

[14] Alfredsson J , Stebbins A , Brennan JM , Matsouaka R , Afilalo J , Peterson ED , et al. Gait speed predicts 30‐day mortality after transcatheter aortic valve replacement: results from the Society of Thoracic Surgeons/American College of Cardiology Transcatheter Valve Therapy Registry. Circulation 2016;133:1351–1359. 26920495 10.1161/CIRCULATIONAHA.115.020279

[15] Cao L , Morley JE . Sarcopenia is recognized as an independent condition by an International Classification of Disease, Tenth Revision, Clinical Modification (ICD‐10‐CM) Code. J Am Med Dir Assoc 2016;17:675–677. 27470918 10.1016/j.jamda.2016.06.001

[16] Studenski SA , Peters KW , Alley DE , Cawthon PM , McLean RR , Harris TB , et al. The FNIH sarcopenia project: rationale, study description, conference recommendations, and final estimates. J Gerontol A Biol Sci Med Sci 2014;69:547–558. 24737557 10.1093/gerona/glu010 PMC3991146

[17] Chen LK , Liu LK , Woo J , Assantachai P , Auyeung TW , Bahyah KS , et al. Sarcopenia in Asia: consensus report of the Asian Working Group for Sarcopenia. J Am Med Dir Assoc 2014;15:95–101. 24461239 10.1016/j.jamda.2013.11.025

[18] Janssen I , Heymsfield SB , Ross R . Low relative skeletal muscle mass (sarcopenia) in older persons is associated with functional impairment and physical disability. J Am Geriatr Soc 2002;50:889–896. 12028177 10.1046/j.1532-5415.2002.50216.x

[19] Baumgartner RN , Koehler KM , Gallagher D , Romero L , Heymsfield SB , Ross RR , et al. Epidemiology of sarcopenia among the elderly in New Mexico. Am J Epidemiol 1998;147:755–763. 9554417 10.1093/oxfordjournals.aje.a009520

[20] Cruz‐Jentoft AJ , Bahat G , Bauer J , Boirie Y , Bruyère O , Cederholm T , et al. Sarcopenia: revised European consensus on definition and diagnosis. Age Ageing 2018;48:afy169‐afy169. 10.1093/ageing/afz046 PMC6593317 31081853

[21] Newman AB , Kupelian V , Visser M , Simonsick EM , Goodpaster BH , Kritchevsky SB , et al. Strength, but not muscle mass, is associated with mortality in the health, aging and body composition study cohort. J Gerontol A Biol Sci Med Sci 2006;61:72–77. 16456196 10.1093/gerona/61.1.72

[22] Cawthon PM , Fox KM , Gandra SR , Delmonico MJ , Chiou CF , Anthony MS , et al. Do muscle mass, muscle density, strength, and physical function similarly influence risk of hospitalization in older adults? J Am Geriatr Soc 2009;57:1411–1419. 19682143 10.1111/j.1532-5415.2009.02366.x PMC3269169

[23] Jung HW , Jang IY , Lee YS , Lee CK , Cho EI , Kang WY , et al. Prevalence of frailty and aging‐related health conditions in older Koreans in rural communities: a cross‐sectional analysis of the Aging Study of Pyeongchang Rural Area. J Korean Med Sci 2016;31:345–352. 26952571 10.3346/jkms.2016.31.3.345 PMC4779857

[24] von Haehling S , Morley JE , Coats AJS , Anker SD . Ethical guidelines for publishing in the Journal of Cachexia, Sarcopenia and Muscle: update. J Cachexia Sarcopenia Muscle 2017 2017;8:1081–1083. 10.1002/jcsm.12261 PMC5700441 29098794

[25] Guralnik JM , Simonsick EM , Ferrucci L , Glynn RJ , Berkman LF , Blazer DG , et al. A short physical performance battery assessing lower extremity function: association with self‐reported disability and prediction of mortality and nursing home admission. J Gerontol 1994;49:M85–M94. 8126356 10.1093/geronj/49.2.m85

[26] Searle SD , Mitnitski A , Gahbauer EA , Gill TM , Rockwood K . A standard procedure for creating a frailty index. BMC Geriatr 2008;8:24. 18826625 10.1186/1471-2318-8-24 PMC2573877

[27] Jung HW , Jang IY , Lee CK , Yu SS , Hwang JK , Jeon C , et al. Usual gait speed is associated with frailty status, institutionalization, and mortality in community‐dwelling rural older adults: a longitudinal analysis of the Aging Study of Pyeongchang Rural Area. Clin Interv Aging 2018;13:1079–1089. 29922046 10.2147/CIA.S166863 PMC5995421

[28] Fried LP , Tangen CM , Walston J , Newman AB , Hirsch C , Gottdiener J , et al. Frailty in older adults: evidence for a phenotype. J Gerontol A Biol Sci Med Sci 2001;56:M146–M156. 11253156 10.1093/gerona/56.3.m146

[29] Won CW , Yang KY , Rho YG , Kim SY , Lee EJ , Yoon JL , et al. The development of Korean activities of daily living (K‐ADL) and Korean instrumental activities of daily living (K‐IADL) scale. J Korean Geriatr Soc 2002;6:107–120.

[30] Park JH , Kim KW . A review of the epidemiology of depression in Korea. J Korean Med Assoc 2011;54:362–369.

[31] Kang Y , Na DL , Hahn S . A validity study on the Korean Mini‐Mental State Examination (K‐MMSE) in dementia patients. J Korean Neurol Assoc 1997;15:300–308.

[32] Rubenstein LZ , Harker JO , Salvà A , Guigoz Y , Vellas B . Screening for undernutrition in geriatric practice developing the short‐form mini‐nutritional assessment (MNA‐SF). J Gerontol A Biol Sci Med Sci 2001;56:M366–M372. 11382797 10.1093/gerona/56.6.m366

[33] Newson RB . Comparing the predictive powers of survival models using Harrell's C or Somers' D. Stata J 2010;10:339–358.

[34] Rosenberg IH . Sarcopenia: origins and clinical relevance. Clin Geriatr Med 2011;27:337–339. 21824550 10.1016/j.cger.2011.03.003

[35] Pinedo‐Villanueva R , Westbury LD , Syddall HE , Sanchez‐Santos MT , Dennison EM , Robinson SM , et al. Health care costs associated with muscle weakness: a UK population‐based estimate. Calcif Tissue Int 2019;104:137–144. 30244338 10.1007/s00223-018-0478-1 PMC6330088

[36] Steffl M , Sima J , Shiells K , Holmerova I . The increase in health care costs associated with muscle weakness in older people without long‐term illnesses in the Czech Republic: results from the Survey of Health, Ageing and Retirement in Europe (SHARE). Clin Interv Aging 2017;12:2003. 29225462 10.2147/CIA.S150826 PMC5708194

[37] Jang I‐Y , Jung H‐W , Park H , Lee CK , Yu SS , Lee YS , et al. A multicomponent frailty intervention for socioeconomically vulnerable older adults: a designed‐delay study. Clin Interv Aging 2018, In press;13:1799–1814. 30275687 10.2147/CIA.S177018 PMC6156114

[38] Guralnik JM , Ferrucci L , Pieper CF , Leveille SG , Markides KS , Ostir GV , et al. Lower extremity function and subsequent disability: consistency across studies, predictive models, and value of gait speed alone compared with the short physical performance battery. J Gerontol A Biol Sci Med Sci 2000;55:M221–M231. 10811152 10.1093/gerona/55.4.m221 PMC12149745

[39] Perera S , Patel KV , Rosano C , Rubin SM , Satterfield S , Harris T , et al. Gait speed predicts incident disability: a pooled analysis. J Gerontol A Biol Sci Med Sci 2016;71:63–71. 26297942 10.1093/gerona/glv126 PMC4715231

[40] Studenski S , Perera S , Patel K , Rosano C , Faulkner K , Inzitari M , et al. Gait speed and survival in older adults. JAMA 2011;305:50–58. 21205966 10.1001/jama.2010.1923 PMC3080184

[41] Jung HW , Kim SW , Ahn S , Lim JY , Han JW , Kim TH , et al. Prevalence and outcomes of frailty in korean elderly population: comparisons of a multidimensional frailty index with two phenotype models. PLoS ONE 2014;9:e87958. 24505338 10.1371/journal.pone.0087958 PMC3913700

[42] Studenski S , Hayes RP , Leibowitz RQ , Bode R , Lavery L , Walston J , et al. Clinical Global Impression of Change in Physical Frailty: development of a measure based on clinical judgment. J Am Geriatr Soc 2004;52:1560–1566. 15341562 10.1111/j.1532-5415.2004.52423.x

[43] Ga H , Won CW , Jung H‐W . Use of the frailty index and FRAIL‐NH scale for the assessment of the frailty status of elderly individuals admitted in a long‐term care hospital in Korea. AnnGeriatr Med Res 2018;22:20–25. 10.4235/agmr.2018.22.1.20 PMC7387636 32743239

